# Preparation and Evaluation of Novel Epitope-Based ETEC K88-K99 Bivalent Vaccine

**DOI:** 10.3390/vetsci12040381

**Published:** 2025-04-18

**Authors:** Shuangshuang Wang, Yuxin Yang, Xinru Yue, Zewen Liu, Fangyan Yuan, Keli Yang, Jiajia Zhu, Wei Liu, Yongxiang Tian, Qiong Wu, Ting Gao, Chang Li, Haofei Song, Danna Zhou, Weicheng Bei

**Affiliations:** 1 National Key Laboratory of Agricultural Microbial Resources Discovery and Utilization, Huazhong Agricultural University, Wuhan 430070, China; wss123@webmail.hzau.edu.cn (S.W.); whisper79@webmail.hzau.edu.cn (X.Y.); 2 The Cooperative Innovation Center for Sustainable Pig Production, Huazhong Agricultural University, Wuhan 430070, China; 3 Hubei Hongshan Laboratory, Huazhong Agricultural University, Wuhan 430070, China; 4 Key Laboratory of Prevention and Control Agents for Animal Bacteriosis (Ministry of Agriculture and Rural Affairs), Hubei Provincial Key Laboratory of Animal Pathogenic Microbiology, Institute of Animal Husbandry and Veterinary, Hubei Academy of Agricultural Sciences, Wuhan 430064, China; yyx20230427@163.com (Y.Y.); liuzwen2004@hbaas.com (Z.L.); fyyuan@hbaas.com (F.Y.); keliy6@hbaas.com (K.Y.); xmszjj@hbaas.com (J.Z.); liuwei@hbaas.com (W.L.); tyxanbit@hbaas.com (Y.T.); wuqiong302@hbaas.com (Q.W.); gaoting2017@hbaas.com (T.G.); lichang1113@hbaas.com (C.L.); 17861509838@163.com (H.S.)

**Keywords:** Enterotoxigenic *Escherichia coli*, FaeG, FanC, epitope, vaccine

## Abstract

Enterotoxigenic *Escherichia coli* (ETEC) is one of the primary pathogens causing diarrhea in piglets, causing significant economic losses in the swine farming industry. Due to the numerous serotypes of ETEC, traditional vaccines fail to provide sufficient cross-protection, and subunit vaccines based on epitope design have emerged as a safer and more effective approach for prevention and control. Unlike vaccine development strategies that involve the tandem arrangement of multiple antigenic epitopes, this study used the K88-FaeG protein as a backbone and incorporated the antigenic epitopes of K99-FanC to achieve a better immunogenicity. By using bioinformatics software to predict B-cell linear epitopes (score of over 0.6), B-cell epitopes from three-dimensional structures (50% amino acid score of ≥0.2), and B-cell epitope IgG antibody subtypes, as well as docking analysis with Sus scrofa aminopeptidase N (APN) receptors, six antigenic epitopes of K99-FanC were selected. Through Western blotting and competitive ELISA, we confirmed that all six recombinant proteins exhibited binding capabilities to K88- and K99-positive serum. The ELISA results showed that the serum levels of specific IgG and IgA antibodies increased after immunization, with FaeG-Ep3 and FaeG-Ep5 inducing the highest antibody titers against FanC-IgG (Log2 = 14.96) and FaeG-IgG (Log2 = 17.96), respectively. Bacterial adhesion assays revealed that only FaeG-Ep3 effectively blocked the adhesion of both K99 and K88 to IPEC-J2 cells. Immunization challenge experiments showed that, in the unimmunized group, mice infected with K88 and K99 experienced weight loss (*p* < 0.05) with intestinal villus shedding and intestinal wall structural damage. However, in the FaeG-Ep3-immunized group, no significant weight loss occurred after infection, and the villus protection rate (83%) was the same as that in the FaeG and FanC immunized groups. Overall, the FaeG-Ep3 recombinant protein identified in this study shows potential vaccine application value and provides new insights for developing multivalent vaccines against ETEC.

## Simple Summary

Enterotoxigenic *Escherichia coli* (ETEC) is a major cause of piglet diarrhea and economic losses in swine farming. Traditional vaccines are limited by insufficient cross-protection due to diverse ETEC serotypes. This study developed a recombinant subunit vaccine by inserting K99-FanC epitopes into the K88-FaeG protein. The key findings include the following: FaeG-Ep3 showed the highest antibody titers and effectively blocked K88 and K99 adhesion; in challenge experiments, FaeG-Ep3 provided protection against intestinal damage. These results indicate that FaeG-Ep3 may serve as a potential vaccine candidate and provide useful insights for the development of multivalent ETEC vaccines.

## 1. Introduction

Diarrhea is common in newborn piglets and weaned piglets. Due to the limited alkalinity and digestive capacity of the gastrointestinal tract at birth, symptoms typically appear 3–5 days after birth. Clinically, it is divided into viral and bacterial diarrhea. The main viral pathogens are porcine epidemic diarrhea virus (PEDV) [1] and transmissible gastroenteritis virus (TGEV) [2], while the main bacterial pathogen is Enterotoxigenic *Escherichia coli* (ETEC) [3]. After ETEC infection, the high morbidity and mortality it induces in piglets cause certain economic losses to the swine industry [4]. Currently, there are multiple strategies to control and prevent postweaning diarrhea (PWD), and vaccination remains a safe and effective means [5]. ETEC can produce adhesins and enterotoxins, which facilitate the colonization of intestinal epithelial cells and stimulate intestinal fluid secretion, respectively, thereby leading to diarrhea. Based on fimbrial adhesin typing, F4 (K88), F5 (K99), F6 (987P), F18, and F41, are associated with ETEC virulence [6,7,8,9,10]. Among these pili, F5, F6, F17, and F41 have been associated with diarrhea in neonatal piglets, and F18 has been associated with diarrhea in weaned piglets. Notably, F4 has been implicated in diarrhea among both newborn and weaned piglets [11]. ETEC pili adhesins play a crucial role in its pathogenesis, and pilus proteins have been shown to be safe and effective as ETEC vaccines [12,13]. Given the numerous challenges in ETEC vaccine development, including strain genomic heterogeneity, antigenic diversity, and the low immunogenicity of key virulence factors such as ST toxins, as well as the issues of co-infections in endemic areas [14] and whole-cell vaccines failing to provide broad-spectrum protection [15], developing a multivalent epitope vaccine based on fimbrial adhesins holds a certain significance for prevention and control.

The prevalence of ETEC is influenced by geographical factors [16]. In surveys of PWD prevalence, K88 strains have emerged as the predominant strains, with an infection rate of 95% [17]. However, in some regions, K99 strains are the predominant cause, with an infection rate of 12% [18]. Among ETEC strains, the K88 strain exhibits the highest prevalence and significant virulence diversity, and it is often found in coinfection with K99 [19]. Therefore, it is of great significance to develop a bivalent epitope vaccine against K88/K99. In the ETEC strain, K88ab, K88ac, and K88ad are three antigenic variants of K88 fimbrial adhesins. K88 fimbriae, consisting primarily of more than 100 repeated FaeG subunits, specifically bind to K88 receptors in the small intestine, resulting in diarrheal symptoms [20,21]. FaeG, a crucial component of fimbrial adhesins, contributes to the sequence divergence observed in K88 fimbrial genes across various serotypes. The primary subunit of K99 fimbriae is encoded by the FanC gene, with each fimbria containing numerous FanC monomers [22]. Multiepitope Fusion Antigen (MEFA) technology is being employed to develop vaccines targeting multiple fimbrial types of ETEC to prevent and manage infections [5]. Exploring epitopes in ETEC is crucial for developing multi-epitope vaccines. For example, effective epitopes have been identified for the K88 fimbrial adhesin FaeG [23,24] and for heat-labile toxin (LT) [25], heat-stable toxin a (STa) [26,27,28], and heat-stable toxin b (STb) [26]. Additionally, neutralizing epitopes of the F18 fimbrial adhesin Fedf have been identified [29]. However, effective epitopes have not been identified for the K99-FanC, and carriers are often used to deliver K99 antigens to induce immune protection [30,31].

The rapid development of bioinformatics and immunoinformatics has made structure-based multi-epitope vaccines one of the most effective applications for vaccine development. The use of bioinformatics to identify antigenic epitopes is a widely recognized method in vaccine development, providing guidance for immunological targets in vaccine design [32,33]. This study aimed to develop a multi-epitope vaccine against K88 and K99 via experimental data and immunoinformatic analysis to select potential antigenic candidate proteins. B-cell epitopes within the K99-FanC subunit were predicted using a computational program and, subsequently, a single epitope was fused onto the K88-FaeG structural subunit. Furthermore, the effectiveness of the developed K88-K99 vaccine was evaluated in a mouse model, demonstrating that immunization with FaeG-Ep3 can alleviate the gut damage caused by K88 and K99 infections, showing a certain degree of protective effect.

## 2. Methods and Materials

### 2.1. Bacterial Strains and Plasmids

The strains and plasmids used in this study are listed in Table 1. The genomic DNA data extracted from ETEC C83549 (K88ac) were used for polymerase chain reaction (PCR) amplification of the FaeG, FaeG-Ep1, FaeG-Ep2, FaeG-Ep3, FaeG-Ep4, FaeG-Ep5, and FaeG-Ep6 genes. Similarly, the genomic DNA data from ETEC C83644 (K99) were used for PCR amplification of the FanC gene. The vector pET30a (+) (Hubei Academy of Agricultural Sciences, Wuhan, China) was used for the cloning of the FaeG, FanC, and FaeG-Ep genes in DH5a cells (Vazyme, Nanjing, China), and the expressions of these genes’ proteins were subsequently induced in BL21 cells (Vazyme, Nanjing, China).

### 2.2. Bioinformatics-Based Prediction of K99-FanC Epitopes and FaeG-Ep Fusion Construction

The Immune Epitope Database (IEDB) is a freely accessible database funded by the National Institutes of Allergy and Infectious Diseases (NIAID). Linear epitopes from the protein sequence and epitopes from the protein structure were predicted using the IEDB database (https://www.iedb.org/, accessed on 28 November 2024). Additionally, the ABCpred Server (https://webs.iiitd.edu.in/raghava/abcpred/ABC_help.html, accessed on 28 November 2024) was used to predict linear B-cell epitopes. The IgPred Server (https://webs.iiitd.edu.in/raghava/igpred/, accessed on 28 November 2024) was used to predict the scores of IgG antibody induced by amino acids. The bioinformatics software DiscoTope-3 (https://biolib.com/DTU/DiscoTope-3, accessed on 16 May 2023) was used to predict B-cell epitopes from three-dimensional structures of K99-FanC. The Molecular Operating Environment molecular graphics system (Chemical Computing Group Inc., Montreal, QC, Canada) was used to display the position of each epitope on the FanC protein. The HDOCK (http://hdock.phys.hust.edu.cn, accessed on 28 January 2025) website was used to predict the docking between the amino acids FaeG and FaeG-Ep and Sus scrofa aminopeptidase N (APN). Proteins and docking sites were visualized using PyMol 3.0 (DeLano Scientific LLC, San Francisco, CA, USA).

Primers containing homologous sequences of the FaeG gene were designed, and overlapping PCR was used to insert the nucleotide sequences encoding the FanC epitope into the 163–173 AA region of the FaeG gene (Table S1). PCR amplification was performed under the following conditions: 30 cycles of denaturation at 94 °C, annealing at 60 °C, and extension at 72 °C. Subsequently, the FaeG epitope fusion gene was digested with *BamH*I and *Not*I restriction enzymes (New England Biolabs, Beijing, China). The amplification of the FaeG and FanC genes was performed similarly to the method described above, with FanC gene digestion performed using *Hind*III and *Xho*I restriction endonucleases (New England Biolabs, Beijing, China). The digested fragment was purified by agarose gel electrophoresis and ligated into the pET-30a (+) vector, which had been similarly digested with the same enzymes. The ligation product was then transformed into *E. coli* DH5α for cloning and storage. Finally, the plasmid was transformed into BL21 cells for protein expression.

### 2.3. Expression and Identification of the FaeG-Ep Fusion Protein and the FaeG and FanC Proteins

Recombinant FaeG, FanC, and FaeG-Ep epitope fusion proteins were expressed in *E. coli* BL21 (DE3). Positive clones were selected based on kanamycin resistance and verified by colony PCR. The selected clones were cultured in kanamycin-containing LB broth at 37 °C with shaking at 200 rpm overnight. The next day, 1% of the overnight culture was inoculated into fresh LB medium and grown until the optical density (OD) at 600 nm reached 0.6–0.8. Protein expression was induced by adding 0.5 mM or 1 mM isopropyl-β-D-thiogalactopyranoside (IPTG). For the first induction condition, the culture was incubated at 37 °C for 6 h. For the second induction condition, the culture was incubated at 16 °C for 16 h. In both conditions, the proteins were expressed in inclusion bodies. Bacteria were harvested by centrifugation at 6000× *g* for 10 min. The proteins were isolated by lysing the BL21 cells, and the inclusion bodies were washed with 4 M urea containing 1% Triton X-100 to remove cellular debris. The inclusion bodies were then purified using sodium N-lauroyl-sarcosinate (sarkosyl/SKL) [34] and subjected to denaturation in 8 M urea, 50 mM Tris-HCl (pH 8.0), and 1 mM DTT. The denatured proteins were refolded by rapid dilution or dialysis in a buffer containing 50 mM Tris-HCl (pH 8.0), with appropriate oxidizing and reducing agents, followed by purification using conventional methods to ensure proper folding and functionality. The refolded fusion proteins were detected by Coomassie Blue staining on sodium dodecyl sulfate–polyacrylamide gel electrophoresis (SDS-PAGE) and characterized by Western blotting using K88 FaeG serum (1:500, *v*/*v*; rabbit serum, Hubei Academy of Agricultural Sciences, Wuhan, China) and K99 FanC serum (1:1000, *v*/*v*; rabbit serum, Hubei Academy of Agricultural Sciences, Wuhan, China).

### 2.4. Enzyme-Linked Immunosorbent Assay (ELISA)

In the competitive ELISA, K99-FanC protein was the coated antigen and rabbit serum K99-FanC was the primary antibody. FaeG-Ep fusion protein and FanC protein acted as competitors, binding to the specific antigen–antibody recognition sites of the K99-FanC rabbit serum and causing a decrease in OD450 values. This method assessed the competition efficiency of each competitor to identify the dominant B-cell epitopes of the K99-FanC antigen. The FaeG-Ep fusion protein (4 μg) was incubated with K99-FanC serum dilutions (1:2000) for 30 min at 37 °C. Each mixture was added to K99-FanC-coated ELISA plate wells (20 ng per well). Following 1 h of incubation at 37 °C, the wells were washed with phosphate-buffered saline containing 0.05% Tween-20 (PBST). The plate was then incubated with horseradish peroxidase (HRP)-conjugated goat anti-rabbit immunoglobulin IgG (1:5000; Proteintech Group, Inc., Wuhan, China). The OD450 was subsequently measured after the addition of 3,3′,5,5′-tetramethylbenzidine (TMB; Vazyme, Nanjing, China) for 10 min at 25 °C. The formula for calculating the relative difference rate is as follows: Relative difference rate (%) = (OD450_(FaeG-Ep)_− OD450_(FanC)_)/OD450_(FanC)_ × 100%.

The serum IgG titers of the FaeG, FanC, and FaeG-Ep fusion proteins were determined by indirect ELISA using mouse sera collected after three immunizations. Briefly, ELISA plates were coated with the optimal antigen concentration and incubated overnight at 4 °C. After antigen fixation, the plates were blocked with 5% (*w*/*v*) skimmed milk in PBS at 37 °C for 2 h. Following three washes with PBST (0.05% Tween-20 in PBS) for 5 min each, serially diluted mouse sera (collected after three immunizations) were added to the wells and incubated at 37 °C for 1 h. The plates were then washed five times with PBST, and HRP-conjugated goat anti-mouse IgG secondary antibody (1:5000; Proteintech Group, Wuhan, China) was added and incubated at 37 °C for 1 h. The ELISA procedures were performed as previously described.

The specific IgA antibody titers in the sera of mice after the third immunization were determined by ELISA. Briefly, ELISA plates were coated with 1 μg/mL of the respective antigen in coating buffer and incubated overnight at 4 °C. After blocking with 5% (*w*/*v*) skimmed milk in PBS at 37 °C for 2 h, the plates were washed three times with PBST (0.05% Tween-20 in PBS). Mouse sera, diluted 1:40, were added to the wells and incubated at 37 °C for 1 h. The plates were then washed five times with PBST, and HRP-conjugated goat anti-mouse IgA secondary antibody (1:2000; Beijing Bosi Biotechnology Co., Ltd., Beijing, China) was added and incubated at 37 °C for 1 h. The ELISA procedures were performed as previously described.

The IgG titers of anti-FaeG and anti-FanC in the serum samples from mice immunized with the FaeG-Ep fusion protein were determined by ELISA on day 42. FaeG and FanC were coated at the antigen concentrations specified in Table 2. The ELISA procedures were performed as previously described, including coating, blocking, serum incubation, washing, secondary antibody incubation, substrate development, and OD450 measurement. Negative control wells containing sera from non-immunized mice were included to establish a baseline. The IgG titers were determined based on the OD450 readings.

ELISA was performed to detect the specific IgG antibody levels of the FaeG-Ep3 antigen against K88- and K99-positive pig sera. The FaeG-Ep3, FaeG, and FanC antigens were coated at a concentration of 4 μg/mL in coating buffer and incubated overnight at 4 °C. After blocking with 5% (*w*/*v*) skimmed milk in PBS at 37 °C for 2 h, the plates were washed three times with PBST (0.05% Tween-20 in PBS). The positive pig sera, which were standard monofactorial sera provided by the Hubei Academy of Agricultural Sciences, Wuhan, China, and diluted 1:20, were added to the wells, followed by incubation at 37 °C for 1 h. Negative pig sera served as the control group. The plates were then washed five times with PBST, and HRP-conjugated goat anti-pig IgG secondary antibody (1:5000; Proteintech Group, Wuhan, China) was added and incubated at 37 °C for 1 h. A ratio of OD450 values of positive to negative sera greater than or equal to 2.1 was used as the standard for positive detection.

### 2.5. Mouse Immunization and Infection

a. Mouse immunization

BALB/c mice (5-week-old females) were randomly assigned to 11 groups (Hubei CDC, Wuhan, China). Eight groups received subcutaneous immunizations with FaeG-Ep fusion protein, FaeG, or FanC (100 μg/dose in complete Freund’s adjuvant for primary immunization), *n* = 12. Booster immunizations with equivalent antigen doses emulsified in incomplete Freund’s adjuvant were administered every two weeks. Three control groups (PBS control, ETEC K88 challenge control, and ETEC K99 challenge control) did not receive antigen treatment, *n* = 6. Serum samples were collected two weeks after primary immunization and two weeks after the final booster immunization and stored at −80 °C until analysis.

b. Inhibition of bacterial adhesion with mouse serum IgG antibodies

The porcine intestinal cell line IPEC-J2 was obtained from the State Key Laboratory of Agricultural Microbiology (Huazhong Agricultural University, Wuhan, China). IPEC-J2 cells were used to evaluate the inhibitory effect of murine-positive serum containing FaeG-Ep fusion proteins on the adhesion of ETEC K88 and K99 bacteria. IPEC-J2 cells were infected with ETEC K88 and K99 at a multiplicity of infection of 1. They were allowed to interact with the murine-positive serum for 1 h at 37 °C and adhere to the IPEC-J2 cells for 2 h, followed by overnight culture at 37 °C on LB agar-coated plates before counting the colonies.

c. Mouse infection

The animal study protocol was reviewed and approved by the Animal Care and Use Committee of the Animal Science and Veterinary Medicine Research Institute, Hubei Academy of Agricultural Sciences. The study adhered to the guidelines set by the committee and followed the “3R” principles (Replacement, Reduction, and Refinement) to ensure the welfare of the experimental animals. ETEC infection experiments were conducted only on the optimal bivalent epitope vaccine group (Ep3), FaeG, and FanC. The remaining immunized mice continued to be housed. ETEC infection was performed as previously described [24,35], with modifications. Seven groups of mice (*n* = 6) were subjected to water restriction for 24 h, followed by the oral administration of 5 g/L of streptomycin for 12 h. After fasting and dehydration for 24 h, the mice were gavaged with 0.2 mL of 0.3% NaHCO3. Thirty minutes later, three groups of mice were gavaged with ETEC K88 (2 × 10^7^ CFU), and three groups were gavaged with ETEC K99 (2 × 10^10^ CFU). One group of mice was left uninfected. Three days post-challenge, jejunal tissues were collected for histopathological examination at Qianbaodu Laboratory, Wuhan, China.

Cecal contents were aseptically collected from six samples, weighed, and resuspended in sterile PBS. After 10-fold serial dilution, 100 μL of each dilution was spread onto agar plates and incubated at 37 °C overnight. Colony-forming units (CFU) were counted, and bacterial load was calculated as CFU·g^−^ using the following formula: CFU·g^−1^ = (X × dilution factor)/cecal content mass (g), where X is the number of colonies. Bacterial counts are expressed as CFU·g^−1^ of cecal contents.

Mouse feces were aseptically collected on day 3, suspended in 500 μL of PBS, and heated at 100 °C for 10 min. Fimbrial-adhesin-specific PCR detection was then performed using the primers listed in Table S2, following the previously described method [36].

Jejunal damage was assessed and scored as follows: 0 = intact and normal villous epithelium; 1 = mild villous edema with epithelial collapse limited to the villous tips; 2 = mild necrosis in the mid-villus region; 3 = moderate necrosis in the mid-villus region with visible crypts; and 4 = severe villous necrosis with loss of epithelial structure.

### 2.6. Statistical Analyses

The relative values between different groups were analyzed via a *t*-test or two-way analysis of variance via GraphPad Prism^®^ 9.0 software (GraphPad Software, San Diego, CA, USA). ns, *p* > 0.05 indicates no significant difference. *, *p* < 0.05 indicates a significant difference. **, *p* < 0.01 indicates a highly significant difference. ***, *p* < 0.001 and *p* < 0.0001 indicate a very highly significant difference.

## 3. Result

### 3.1. Selection of K99 Immunodominant B-Cell Epitopes

**Figure 1:**
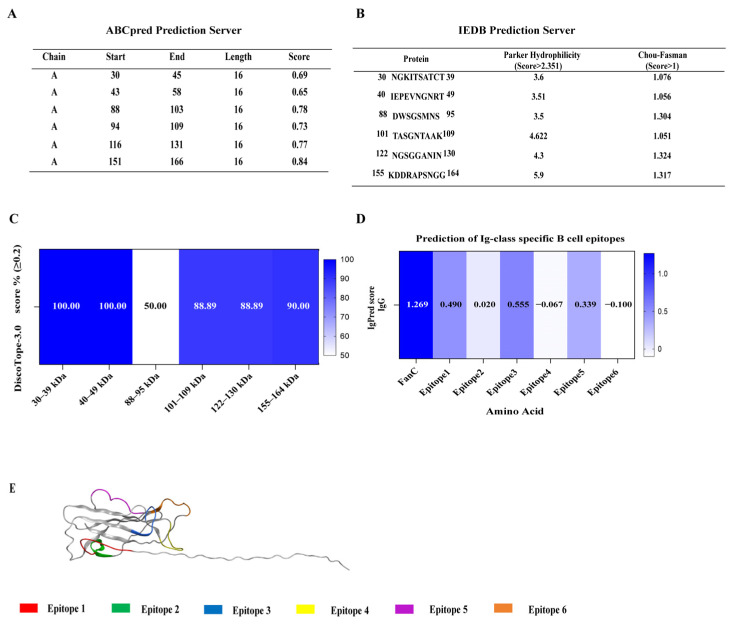
Prediction of B-cell epitopes in ETEC K99-FanC. (**A**): The ABCpred software predicts continuous (linear) B-cell epitopes; (**B**): Parker hydrophilicity and Chou–Fasman algorithms from the IEDB database are used to predict B-cell epitopes; (**C**): the DiscoTope-3 software predicts three-dimensional conformational B-cell epitopes; (**D**): the IgPred software predicts the scores of IgG antibody produced by B-cell epitopes; and (**E**): FanC B-cell epitope displayed on three-dimensional structure, FanC PDB ID: P18103.1.A.

Six epitopes of the K99 FanC antigen were predicted in this study, and all were found to have a good antigenicity (Table 3). In the prediction process, ABCpred software (version 2.0, online version) (score of >0.6) was used to predict continuous (linear) B-cell epitopes with a length of 16mer, and six epitopes were obtained (Figure 1A). The epitope vaccine, based on the K88 FaeG structure, should ensure the stability of the chimeric protein. The B-cell epitope size was designed to be 8-10 AA. Subsequently, based on the prediction results in Figure 1A, the Parker hydrophilicity algorithm (score of >2.351) based on amino acid hydrophilicity and the Chou–Fasman algorithm (score of >1) based on β-angle frequency (Figure 1B) from the IEDB database were used to predict B-cell epitopes, while DiscoTope-3 software was used to predict B-cell epitope (50% amino acid score of ≥0.2) based on the three-dimensional structure of amino acids (Figure 1C). Finally, six epitopes were selected to construct epitope vaccines. The scores for inducing IgG antibody were predicted using IgPred for all six epitopes, as shown in Figure 1D. Epitopes 1, 3, and 5 exhibited the highest IgG scores among them, implying that these might be the preponderant antigenic epitopes. All six epitopes of FanC were either continuous or discontinuous and were present on the protein surface (Figure 1E).

### 3.2. Design of Peptide-Based Protein and Docking Analysis with Sus Scrofa Aminopeptidase N (APN) Receptors

**Figure 2:**
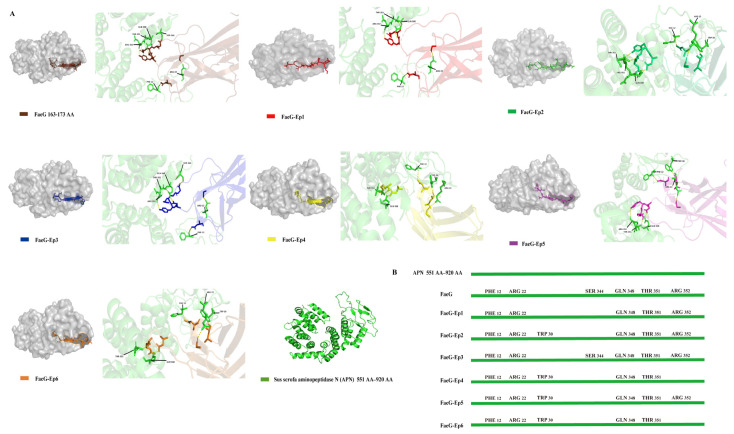
Docking studies of vaccine protein and APN receptors. (**A**): Display of 163–173AA and FaeG epitopes on the three-dimensional structure of FaeG. Molecular docking of FaeG and FaeG-Ep with APN (551 AA–920 AA) receptors analyzed by HDOCK software, FaeG PDB ID: 2j6g.1 and APN PDB ID: 5lds.1.A. (**B**): APN amino acid docking site.

This study involves directly replacing the amino acid residues at positions 163–173 of the FaeG gene with the FanC epitope gene (FaeG-Ep) to design a novel bivalent epitope vaccine. K88-FaeG residues 163–173 do not belong to the FaeG neutralizing epitope [23] and are exposed on the surface of the protein. Fusing the FanC epitope with the 163–173 AA of FaeG causes conformational changes in the 3D structure of the FaeG protein. APN (551 AA–920 AA) is the host receptor protein for the F4 bacterial fimbrial [37]. Docking analyses were performed between APN (551 AA–920 AA) and both the FaeG and FaeG-Ep proteins to predict whether the FaeG structure in the FaeG-Ep fusion antigen was stable (Figure 2A). Compared with the FaeG-APN and FaeG-Ep-APN complexes, only the FaeG-Ep3-APN composite had Ser344 residues, consistent with the FaeG-APN composite (Figure 2B). The prediction results show that Ep3 had no effect on the FaeG protein structure.

### 3.3. Plasmid Construction and Expression of the FaeG-Ep Protein

**Figure 3:**
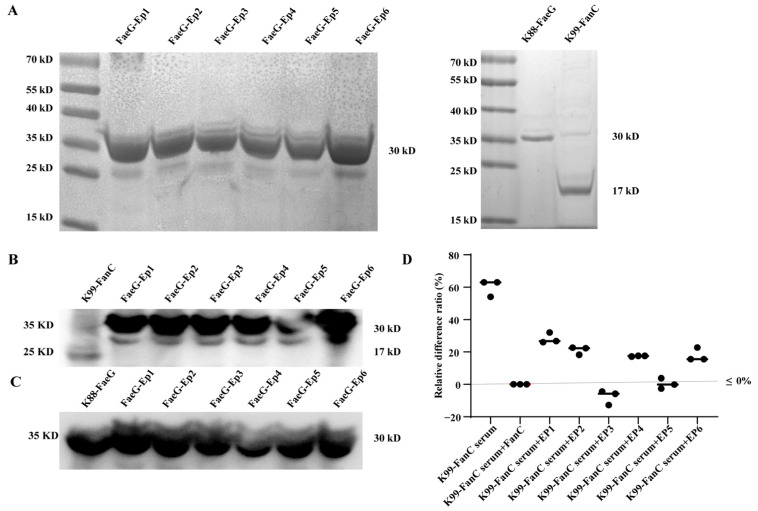
Expression and identification of recombinant proteins. (**A**): SDS-PAGE analysis of FaeG-Ep fusion proteins, FaeG, and FanC expression; (**B**): Western blot analysis of FaeG-Ep fusion proteins and FanC expression; (**C**): Western blot analysis of FaeG-Ep fusion proteins and FaeG expression; and (**D**): ELISA competition assay was conducted utilizing K99-FanC as the encapsulated antigen and each FaeG-Ep protein as the competitor, with anti-K99 serum diluted from 1:2000.

Using PET-30a (+) as a vector, we successfully constructed expression vectors for the K88-FaeG, K99-FanC, and FaeG-Ep fusion proteins. These proteins were successfully expressed in BL21 *E. coli* and purified through a denaturation and renaturation process. SDS-PAGE analysis confirmed successful protein expression (Figure 3A), and specific reactions were verified using K99 FanC (Figure 3B) and K88 FaeG rabbit sera (Figure 3C). In a competitive ELISA with FanC as the coating antigen, the results showed that the FaeG-Ep3 and FaeG-Ep5 proteins could compete with the FanC protein for binding to the FanC serum. Calculations using a relative difference formula (based on the OD450 readings from the FanC protein wells) indicated that only the wells containing individual FaeG-Ep3 or FaeG-Ep5 fusion proteins exhibited a relative difference rate close to 0%, similar to that of the FanC competitor (Figure 3D). These results suggest that Ep3 and Ep5 may be dominant antigenic epitopes of FanC.

### 3.4. Verification of the Effective Antigenic Epitope of FanC-Ep on the K88-FaeG-Ep Vaccine In Vitro

**Figure 4:**
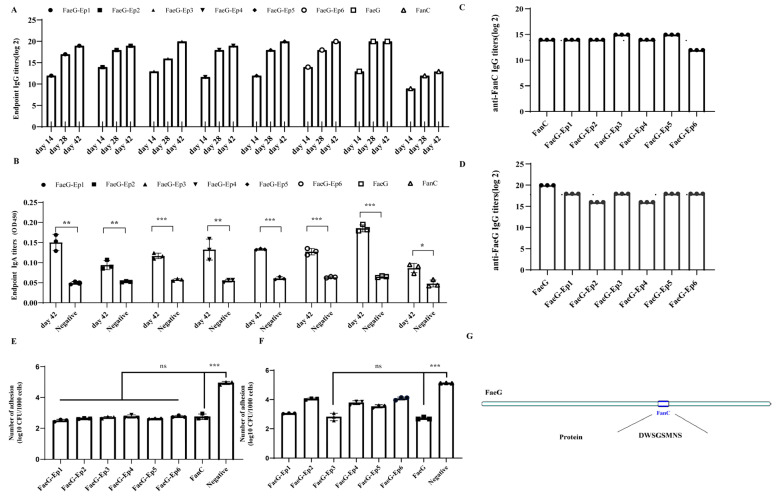
Preparation of FaeG-Ep mouse immune serum and identification of FanC-B-cell epitopes. (**A**): ELISA detection of the FaeG-Ep, FaeG, and FanC sera at 14, 28, and 42 days specific IgG titer; (**B**): ELISA detection of the FaeG-Ep, FaeG, and FanC sera at 42 days specific IgA titer; (**C**): ELISA detection of FaeG-Ep serum specific anti-FanC IgG titer; (**D**): ELISA detection of FaeG-Ep serum specific anti-FaeG IgG titer; (**E**,**F**): neutralization experiment of anti ETEC K99 and ETEC K88 bacterial adhesion to IPEC-J2 cells; and (**G**): the chimeric schematic of the bivalent epitope vaccine FaeG-Ep3. All experimental results are based on three sets of repeated data.

Herein, the mice were subcutaneously immunized with FaeG, FanC, and FaeG-Ep to generate specific antibodies. The cross-chessboard method was used to determine the optimal concentration of antigen coating (Table 2), and the potency of mouse serum was assessed at 14, 28, and 42 days post-immunization. Notably, as the number and duration of immunizations increased, the serum IgG antibody titer gradually increased (Figure 4A). Additionally, specific IgA antibody levels in the sera of mice from all groups were significantly activated on day 42 (Figure 4B). The ELISA results showed that when K99-FanC was used as the coating antigen, FaeG-Ep3 and FaeG-Ep5 had the highest serum titers among the six FaeG-Ep proteins, with IgG antibody titers of 14.96 (log2), higher than the 13.96 (log2) in the K99 FanC serum (Figure 4C). This was likely due to the K88-FaeG backbone protein enhancing the antibody levels against FanC B-cell epitopes [5]. The ELISA assay using K88-FaeG as the coating antigen revealed that the FaeG-Ep3 and FaeG-Ep5 serum IgG titers exhibited the highest IgG titers among the six FaeG-Ep protein serum titers (Figure 4D). This finding shows that Ep3 and Ep5 are the major antigenic epitopes of K99 FanC, without interfering with antibody production against the FaeG protein. Anti-ETEC K88 and anti-ETEC K99 adhesion experiments with different FaeG-Ep sera on IPEC-J2 cells revealed that FanC serum significantly inhibited ETEC K99 adhesion (*p* < 0.001), and the inhibitory effect of FaeG-Ep serum was not significantly different from that of FanC serum (*p* > 0.05) (Figure 4E). FaeG serum significantly inhibited ETEC K88 adhesion, but only FaeG-Ep3 serum showed no significant difference in inhibitory effect compared to FaeG serum (*p* > 0.05) (Figure 4F). FaeG-Ep3 serum could resist the in vitro adhesion of both the ETEC K99 and K88 strains. Ep3 is the optimal epitope for a novel bivalent subunit vaccine against ETEC K88-K99 (Figure 4G). Furthermore, tests showed that the FaeG-Ep3 protein remained stable after 30 days of storage at both −20 °C and −80 °C (Figure S1), demonstrating that FaeG-Ep3 is a stable protein.

### 3.5. Verification of the Effective Antigenic Epitope of FanC-Ep in the K88-FaeG-Ep Vaccine In Vivo

**Figure 5:**
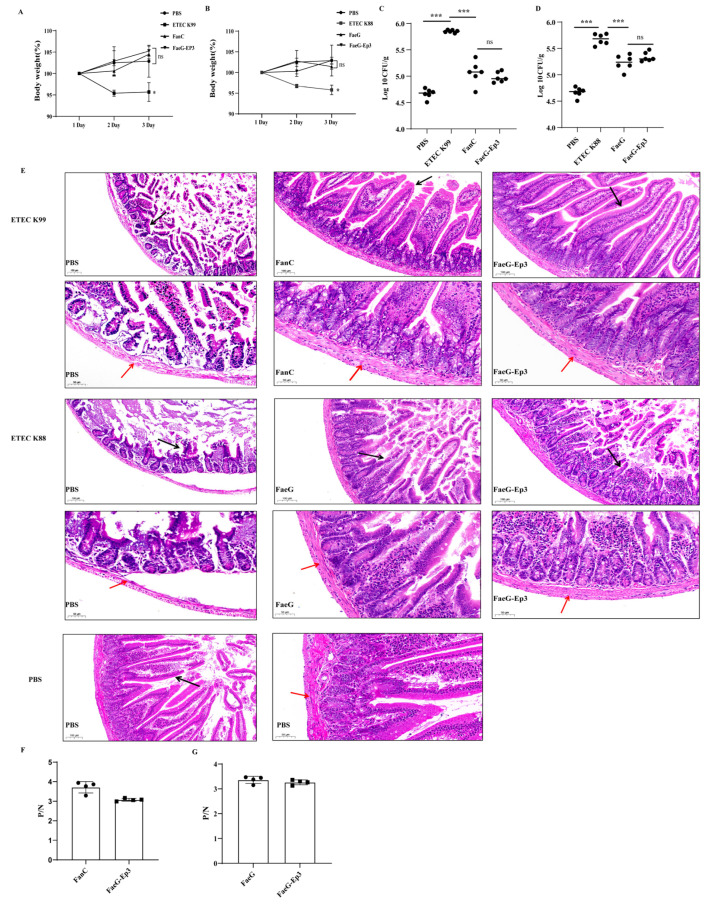
The protective effect of FaeG-Ep antigen on mice. (**A**,**B**): The measurement results of the body weight of mice infected with ETEC K99 and ETEC K88 for three days and (**C**,**D**): bacterial counts performed on the cecal contents of all groups three days post-infection with ETEC K88 and ETEC K99. (**E**): Protective effects of Ep3 antigens on intestinal villi and intestinal mucosal structures in mice infected with ETEC K99 and ETEC K88. Black arrows: intestinal villi; Red arrows: muscular layer (bar = 100 μm and 50 μm). (**F**,**G**): ELISA was used to detect IgG antibodies against FaeG and FaeG-Ep3 proteins in K99 and K88 pig serum.

In this study, we adhered to the 3R principles (Reduction, Refinement, and Replacement) of animal ethics. Subsequent ETEC infection experiments were conducted only on the optimal bivalent epitope vaccine (Ep3), FaeG, and FanC groups. BALB/c mice were infected with ETEC K88 and ETEC K99 following fusion protein immunization. Following a 72 h period, the mice were euthanized for further analysis. Compared to the PBS group, the mice in the ETEC K88 (*p* < 0.05) and ETEC K99 (*p* < 0.05) infection groups showed significant weight loss, while immunized mice exhibited no significant difference in weight (Figure 5A,B). Compared to the PBS group, mice infected with ETEC K88 (*p* < 0.001) and ETEC K99 (*p* < 0.001) showed a significant increase in bacterial load in their cecal contents. In contrast, immunized mice had a significantly lower bacterial load in their cecal contents compared to the infected group (*p* < 0.001) (Figure 5C,D). Mice feces collected at 72 h were analyzed via FaeG- and FanC-specific PCR. The analysis revealed that, compared to the infected group, the immunized group showed less distinct target bands, indicating minimal ETEC colonization (Figure S2). These results indicate that protein immunization reduces ETEC colonization in the small intestine. Jejunum tissue was observed to evaluate the protective effect of Ep3. The control group exhibited neatly arranged intestinal villi, normal muscular layers, and a clear intestinal wall structure. In contrast, the ETEC K88 and K99 infection groups showed complete shedding of the intestinal villi, thinning of the muscular layer, and a compromised intestinal wall structure. Compared with the ETEC K99 infection group, the FanC and FaeG-Ep3 groups exhibited no obvious shedding of the intestinal villi, normal muscular layers, and clear intestinal wall structures. Compared with the ETEC K88 infection group, the FaeG and FaeG-Ep3 groups showed less intestinal villus damage, normal muscle layers, and clear intestinal wall structures (Figure 5E). The protection rate of FaeG-Ep3 was calculated based on the number of mice with jejunal villus shedding, and it was found to be 83% against both ETEC strains following FaeG-Ep3 immunization (Figure S3, Table S3 and S4). The pathological scoring of jejunal tissue was performed using a double-blind method. Compared to the PBS group, significant damage was observed in the ETEC K99 (*p* < 0.01) and ETEC K88 (*p* < 0.001) infection groups. The FaeG-Ep3 protein protected the integrity of intestinal villi and mucosa from damage caused by ETEC K99 (*p* < 0.01) and ETEC K88 (*p* < 0.01) (Figure S4). ELISA results using K99-positive pig serum (Figure 5F) and K88-positive pig serum (Figure 5G) showed that the FaeG-Ep3 protein generated specific reactions with both sera.

## 4. Discussion

To date, vaccination is considered to be a successful long-term strategy for preventing and managing ETEC infections [3]. Usually, piglets acquire passive immunity against neonatal diarrhea through the sow’s colostrum [38]. Developed ETEC vaccines include monovalent F4 live vaccines targeting F4-ETEC [39], a bivalent F4/F18 live vaccine [40], an ETEC ghost inactivated vaccine [41], an LT-LTa-STb-Stx2e vaccine [26] based on the structure of LT toxin, and a FedF-FiG-STb-Stx2e-Sta-LTa vaccine [42]. In addition to whole-cell vaccines and the abovementioned enterotoxin-related vaccines, fimbrial adhesins are also important antigens in the development of ETEC vaccines. Peptide vaccines based on dominant antigenic epitopes are small and simple in molecular structure, have a strong specificity, and are safer and more effective than traditional vaccines. Immunoinformatics tools can identify effective epitopes for vaccine design, saving time and cost compared to traditional vaccine development [43]. This study used bioinformatics software such as IEDB, ABCpred, IgPred, and HDOCK servers to predict six B-cell epitopes of FanC to prepare an effective bivalent epitope vaccine comprising FaeG and FanC.

Vaccine delivery and adjuvant strategies can enhance the immune response required for epitope vaccines. *Salmonella* species [44], *Lactococcus casei* [24,45,46], and recombinant adenovirus vectors have been employed as vaccine delivery vehicles, and the expressed K99 fimbrial proteins have demonstrated effective immunological outcomes. Since specific sequences within the K88ab fimbrial subunit protein can be deleted or copied [21], K88 fimbriae can be used as a display system, exhibiting advantages in enhancing antibody levels and promoting humoral and cellular immunity [47]. Our results revealed the novel chimeric expression of B-cell epitopes on the PET-30a (+) vector using the K88-FaeG subunit as a display system. After the FanC-Ep epitope (163-173AA) was embedded in K88-FaeG, the chimeric FaeG-Ep protein could be recognized by K88- and K99-positive sera, with Ep3 and Ep5 showing the strongest competitive ability against K99-positive serum (Figure 3). Mice immunized with the FaeG-Ep recombinant protein exhibited a significant elevation of their serum IgG and IgA titers. Ep3 and Ep5 mouse immune sera both showed high-titer specific IgG antibodies against FanC (2^14.96^) and were identified as dominant B-cell epitopes of FanC. In this study, the major epitope terminated at 390 bp, differing from the dominant epitopes in the carboxyl terminus of the reported FanC gene [45] and amino acids 418 to 612 [48]. Bacterial neutralization assays showed that only Ep3 mouse serum exhibited anti-adhesion activity against both ETEC K88 and ETEC K99 (Figure 4). This may be related to the fact that only FaeG-Ep3 maintains normal antigenic docking sites after binding to APN (Figure 2). APN mediates K88 adhesion to IPEC-J2 cells [49] and is associated with the immune response induced by K88 pili [37].

Currently, studies have shown that pathogenic *E. coli* can colonize mice. After pretreatment with streptomycin, mice were colonized by Salmonella Typhimurium, resulting in diarrhea [50]. ETEC has been reported to colonize the mouse intestine [51,52], and existing studies have established diarrhea infection models for ETEC K88 [35] and K99 [24]. Based on the above research, this study established an ETEC K88 and K99 infection model using streptomycin-pretreated mice. After infecting the mice with different doses of ETEC K88 and ETEC K99, there was a marked decrease in mental state, significant weight loss, and, in some cases, fecal soiling and even death. Finally, the mice were infected with ETEC K88 (2 × 10^7^ CFU) and ETEC K99 (2 × 10^10^ CFU) to evaluate the protective effects of FaeG-Ep3. After ETEC K99 infection, the villus protection rate in the FaeG-Ep3 immunized group for jejunal tissue was 83%, which is similar to the 90% protection rate reported by Deng [30]. After ETEC K88 infection, the villus protection rate in the FaeG-Ep3 immunized group for jejunal tissue was 83%, which is similar to the 80% protection rate reported by Yu [53]. FaeG-Ep3 exhibits similar protective effects to the FanC and FaeG antigens, preventing ETEC K88 and K99 from colonizing the small intestine and mitigating ETEC-induced villous damage (Figure 5).

Currently, K88-FaeG serves as a scaffold for fusing multiple epitopes, including Fim41a-FanC-FasA [54] and FaeG-FedF-FanC-FasA-Fim41a [5]. FaeG scaffold protein is used for the development of multi-epitope vaccines, and the prokaryotically expressed protein still exhibits a stable immunogenicity. The specific recognition reactions of the FaeG-Ep3 protein with K88 and K99 pig sera indicate that the FaeG-Ep3 protein has a certain immunogenicity and the potential for vaccine development (Figure 5).

The FaeG-Ep3 protein prepared in this study demonstrated certain protective effects and vaccine potential; however, its insolubility limits mass production and direct clinical application. An alternative approach is to utilize vaccine delivery platforms to express FaeG-Ep3 in a more functional and soluble form. For instance, the ETEC K88ac strain has been employed as a vaccine vector, incorporating LT and STa epitopes into the FaeG subunit at amino acid positions 95–105 and 114–122 AA, respectively [55]. This system retains a native-like protein conformation, which may support proper epitope presentation. Another promising platform involves ferritin nanocages, which offer advantages such as a high structural stability, biocompatibility, enhanced immune response, and improved solubility in prokaryotic expression systems [56,57]. Improving the soluble expression of the FaeG-Ep3 protein remains a key objective for future work. In addition to evaluating different expression systems and optimizing soluble production, in vitro experiments using target animal cells will be conducted to assess cell-specific toxicity and uptake kinetics. Further studies will also aim to evaluate its protective efficacy in piglet models.

Our study, using bioinformatics prediction, identified a novel chimeric site where replacing the 163–173 AA in the FaeG protein ensured stability and enhanced immunogenicity. This modification significantly improved protection against ETEC K99 and overcame the low immunogenicity and poor stability issues associated with peptide vaccines. Taken together, the K99-FanC antigenic epitope fusion design, which is based on the structural characteristics of K88-FaeG, presents a potentially effective strategy for developing a broad-spectrum anti-ETEC vaccine.

## 5. Conclusions

Overall, the Ep3 (^88^DWSGSMNS^95^) epitope derived from K99-FanC, after being fused with the heterologous carrier FaeG protein, exhibited antigenicity and inhibited FanC antigen–antibody complex binding. Additionally, FaeG-Ep3 serum could bind FanC antigen and inhibit ETEC K99 and K88 adhesion to IPEC-J2 cells. Following ETEC K99 and K88 infection, Ep3 effectively maintained the structural integrity of mouse jejunal tissues. The findings of this study indicate the potential of FaeG-Ep3 in the development of vaccines targeting ETEC K88 and K99.

## Figures and Tables

**Table 1:** The strains and plasmid information in the study.

Strains	Relevant Properties	Sources
C83549	Porcine *E. coli* field isolate, K88ac	Hubei Academy of Agricultural Sciences
C83644	Porcine *E. coli* field isolate, K99	
pET/FaeG	FaeG + pET30α (+) in DH5α/BL21	This study
pET/FanC	FanC + pET30α (+) in DH5α/BL21	This study
pET/FaeG-Ep1	FaeG-Ep1 + pET30α (+) in DH5α/BL21	This study
pET/FaeG-Ep2	FaeG-Ep2 + pET30α (+) in DH5α/BL21	This study
pET/FaeG-Ep3	FaeG-Ep3 + pET30α (+) in DH5α/BL21	This study
pET/FaeG-Ep4	FaeG-Ep4 + pET30α (+) in DH5α/BL21	This study
pET/FaeG-Ep5	FaeG-Ep5 + pET30α (+) in DH5α/BL21	This study
pET/FaeG-Ep6	FaeG-Ep6 + pET30α (+) in DH5α/BL21	This study

**Table 2:** ELISA antigen coating concentration.

Antigen Name	Concentration	*p*-Value	N-Value	*p*-Value/N-Value
K88-FaeG	11.7 ng/mL	1.18	0.12	9.86
K99-FanC	93.75 ng/mL	1.01	0.06	15.78
FaeG-Ep1	1 μg/mL	1.0	0.07	14.23
FaeG-Ep2	46.87 ng/mL	1.12	0.15	7.36
FaeG-Ep3	4 μg/mL	0.95	0.09	10.23
FaeG-Ep4	4 μg/mL	1.0	0.09	10.23
FaeG-Ep5	4 μg/mL	0.93	0.06	13.63
FaeG-Ep6	187.5 ng/mL	1.0	0.09	11.68

**Table 3:** K88 and K99 fimbrial adhesin subunits and the B-cell epitope of K99-FanC subunit.

Antigen Name	Protein Sequence
K88	FaeG
K99	FanC
Epitope 1	^30^NGKITSATCT^39^
Epitope 2	^40^IEPEVNGNRT^49^
Epitope 3	^88^DWSGSMNS^95^
Epitope 4	^101^TASGNTAAK^109^
Epitope 5	^122^NGSGGANIN^130^
Epitope 6	^155^KDDRAPSNGG^164^

## Data Availability

The datasets used and/or analyzed during the current study are available from the corresponding author on reasonable request.

## Supplementary Materials

The following supporting information can be downloaded at: https://www.mdpi.com/article/10.3390/vetsci12040381/s1. Table S1: Primers used for Overlap PCR to construct FaeG-Ep fusion genes. Table S2: The PCR primer sequences for identification of ETEC. Table S3: Pathological statistics after ETEC K99 infection. Table S4: Pathological statistics after ETEC K88 infection. Figure S1: SDS-PAGE analysis of FaeG-Ep3 protein stability at −80°C and −20°C. Figure S2: Gene-specific PCR detection of FanC and FaeG. Figure S3: Histopathological analysis of the jejunum after ETEC infection. Figure S4: Double blind pathological scoring method.
