# Supplementary material for: Preparation and Evaluation of Novel Epitope-Based ETEC K88-K99 Bivalent Vaccine
Source: Vet Sci. 2025 Apr 18;12(4):381. doi: 10.3390/vetsci12040381 (PMC12030781; doi:10.3390/vetsci12040381)
Supplement: Supplementary file 1 [file vetsci-12-00381-s001.zip › vetsci-3484386 supplementary.pdf]

Preparation and Evaluation of a Novel Epitope-Based ETEC K88-K99 Bivalent Vaccine

Shuangshuang Wang 1,2,3,4; Yuxin Yang 4; Xinru Yue 1,2,3,4; Zewen Liu 4; Fangyan Yuan 4; Keli Yang 4; Jiajia Zhu 4; Wei Liu 4; Yongxiang Tian 4; Qiong wu 4; Ting Gao 4; Chang Li 4; Haofei Song 4; Danna Zhou 4,* and Weicheng Bei 1,2,3,**

1. National Key Laboratory of Agricultural Microbial Resources Discovery and Utilization, Huazhong Agricultural University, Wuhan 430070, China; wss123@webmail.hzau.edu.cn (S.W.); whisper79@webmail.hzau.edu.cn (X.Y.)

2. The Cooperative Innovation Center for Sustainable Pig Production, Huazhong Agricultural University, Wuhan 430070, China

3. Hubei Hongshan Laboratory, Huazhong Agricultural University, Wuhan 430070, China

4. Key Laboratory of Prevention and Control Agents for Animal Bacteriosis (Ministry of Agriculture and Rural Affairs), Hubei Provincial Key Laboratory of Animal Pathogenic Microbiology, Institute of Animal Husbandry and Veterinary, Hubei Academy of Agricultural Sciences, Wuhan 430064, China;

yyx20230427@163.com (Y.Y.); liuzwen2004@hbaas.com (Z.L.); fyyuan@hbaas.com (F.Y.); keliy6@hbaas.com (K.Y.); xmszjj@hbaas.com (J.Z.); liuwei@hbaas.com (W.L.); tyxanbit@hbaas.com (Y.T.); wuqiong302@hbaas.com (Q.W.); gaoting2017@hbaas.com (T.G.); lichang1113@hbaas.com (C.L.); 17861509838@163.com (H.S.)

* Correspondence: zdn66@hbaas.com (D.Z.); beiwc@mail.hzau.edu.cn (W.B.)

Supplemental Table S1. Primers used for Overlap PCR to construct FaeG-Ep fusion genes.

Primer	Sequence(5′ to 3′)	Product size，bp
*BamH*I-FaeG-F	CTGGATCCTGGATGACTGGTGATTTCAA	786
*Not*I-FaeG-R	ATAAGAATGCGGCCGCGTAATAAGTTATTGCTACGTTCAG	
*Hind*III-FanC-F	CAAGCTTTTATCTTAGGTGGTATGG	477
*Xho*I-FanC-R	ACGTCTCGAGTTACATATAAGTGACTAAG	
FaeG-Ep1-AR	TGTACAAGTAGCACTCGTTATTTTGCCATTAGTAACCCCACCTCTCCCTA	783
FaeG-Ep1-BF	AATGGCAAAATAACGAGTGCTACTTGTACAGCCGACGGGTTGAGC	
FaeG-Ep2-AR	GTACGATTACCATTGACCTCAGGCTCAATAGTAACCCCACCTCTCCCTA	783
FaeG-Ep2-BF	ATTGAGCCTGAGGTCAATGGTAATCGTACAGCCGACGGGTTGAGC	
FaeG-Ep3-AR	ACTGTTCATAGAACCAGACCAGTCAGTAACCCCACCTCTCCCTA	777
FaeG-Ep3-BF	GACTGGTCTGGTTCTATGAACAGTGCCGACGGGTTGAGC	
FaeG-Ep4-AR	TTTAGCAGCAGTATTTCCTGAAGCTGTAGTAACCCCACCTCTCCCTA	780
FaeG-Ep4-BF	ACAGCTTCAGGAAATACTGCTGCTAAAGCCGACGGGTTGAGC	
FaeG-Ep5-AR	ATTAATATTAGCACCACCAGACCCATTAGTAACCCCACCTCTCCCTA	780
FaeG-Ep5-BF	AATGGGTCTGGTGGTGCTAATATTAATGCCGACGGGTTGAGC	
FaeG-Ep6-AR	CCACCATTAGACGGAGCGCGGTCATCTTTAGTAACCCCACCTCTCCCTA	783
FaeG-Ep6-BF	AAAGATGACCGCGCTCCGTCTAATGGTGGAGCCGACGGGTTGAGC	

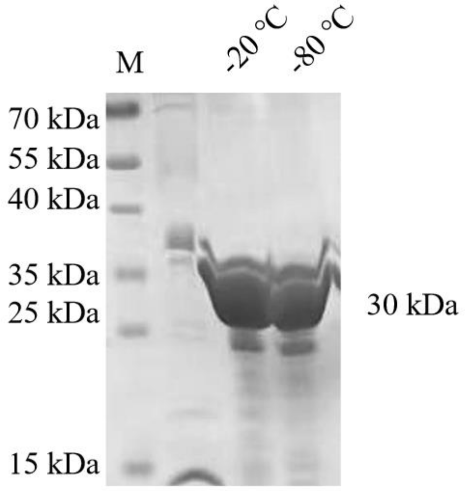

Figure S1. SDS-PAGE analysis of FaeG-Ep3 protein stability at -80°C and -20°C.

Supplemental Table S2. The PCR primer sequences for identification of ETEC

Primer	Sequence(5′ to 3′)	Product size，bp
K88-F	TGGTAGTATCACTGCAGAT	343
K88-R	CACTTTCACTGAACCAACT	
K99-F	GCTCGTATTGACTGGTCT	157
K99-R	CAGCCGTAGTGAATGAAG	

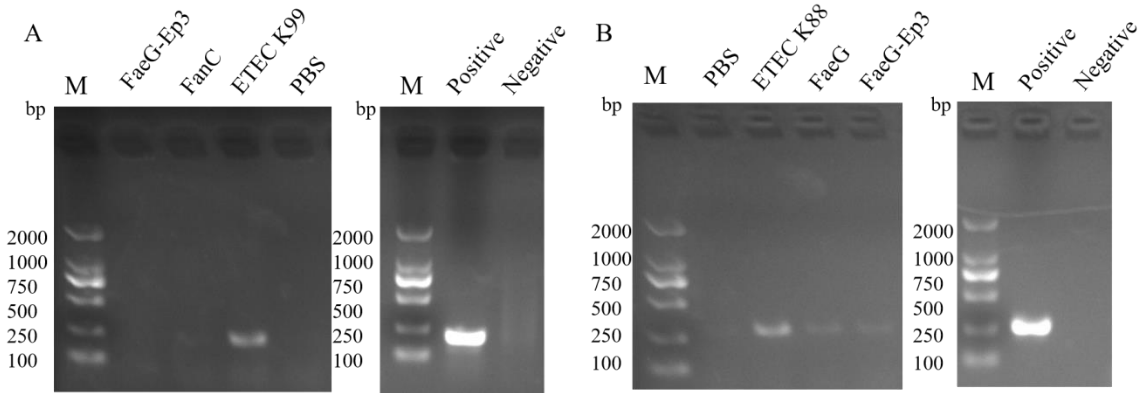

Figure S2. Gene-specific PCR detection of FanC and FaeG.

(A) Detoxification testing after ETEC K99 infection; (B) Detoxification testing after ETEC K88 infection

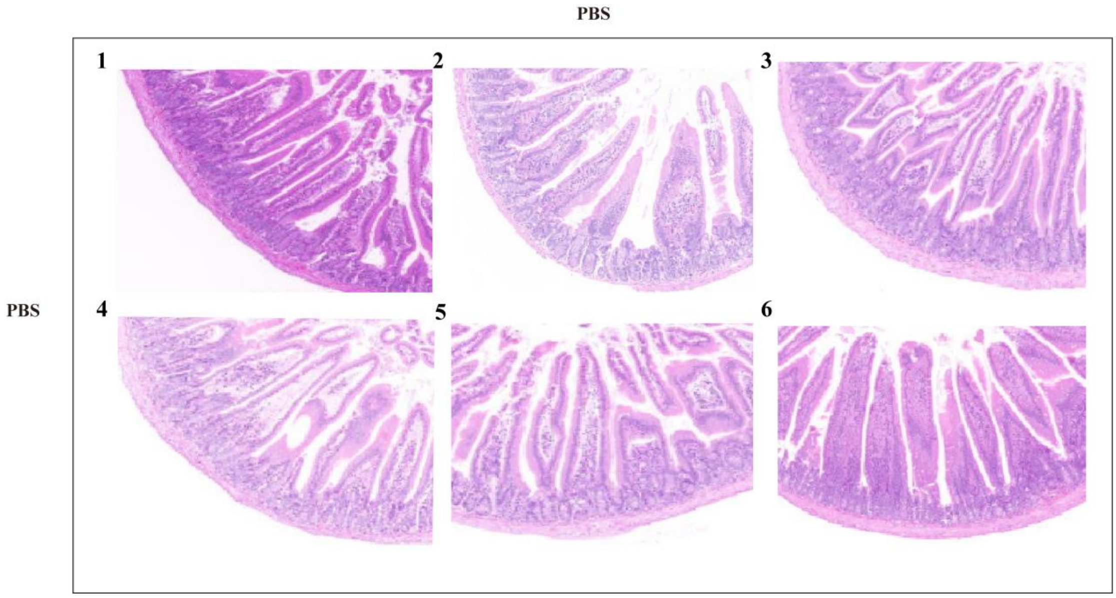

Figure S3. Histopathological analysis of the jejunum after ETEC infection.

(A) PBS control group. bar = 100 μm

Black numbers: No villous shedding.

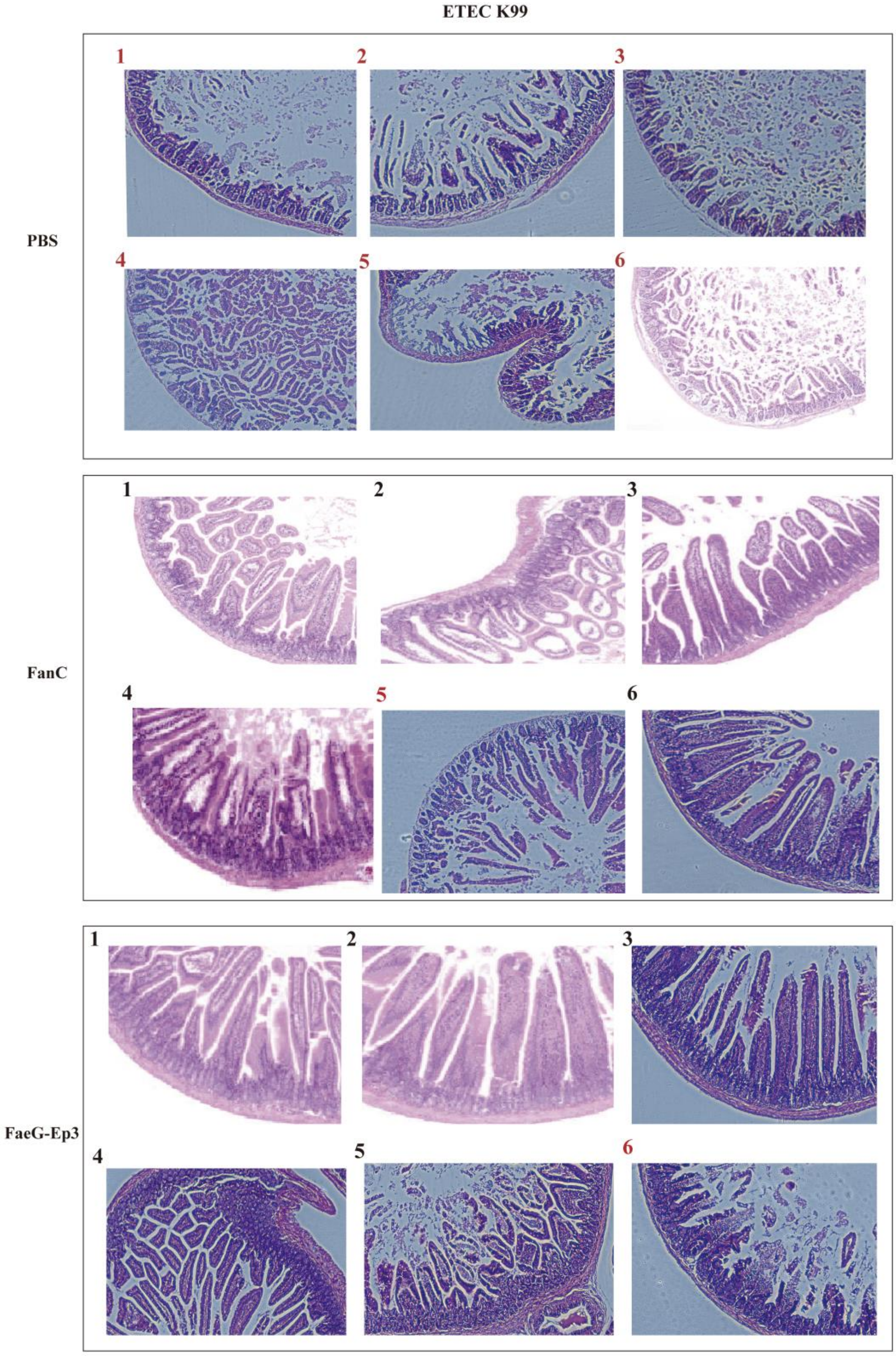

Figure S3. Histopathological analysis of the jejunum after ETEC infection.

(B) ETEC K99 infection group. bar = 100 μm

Red numbers: Villous shedding; black numbers: No villous shedding.

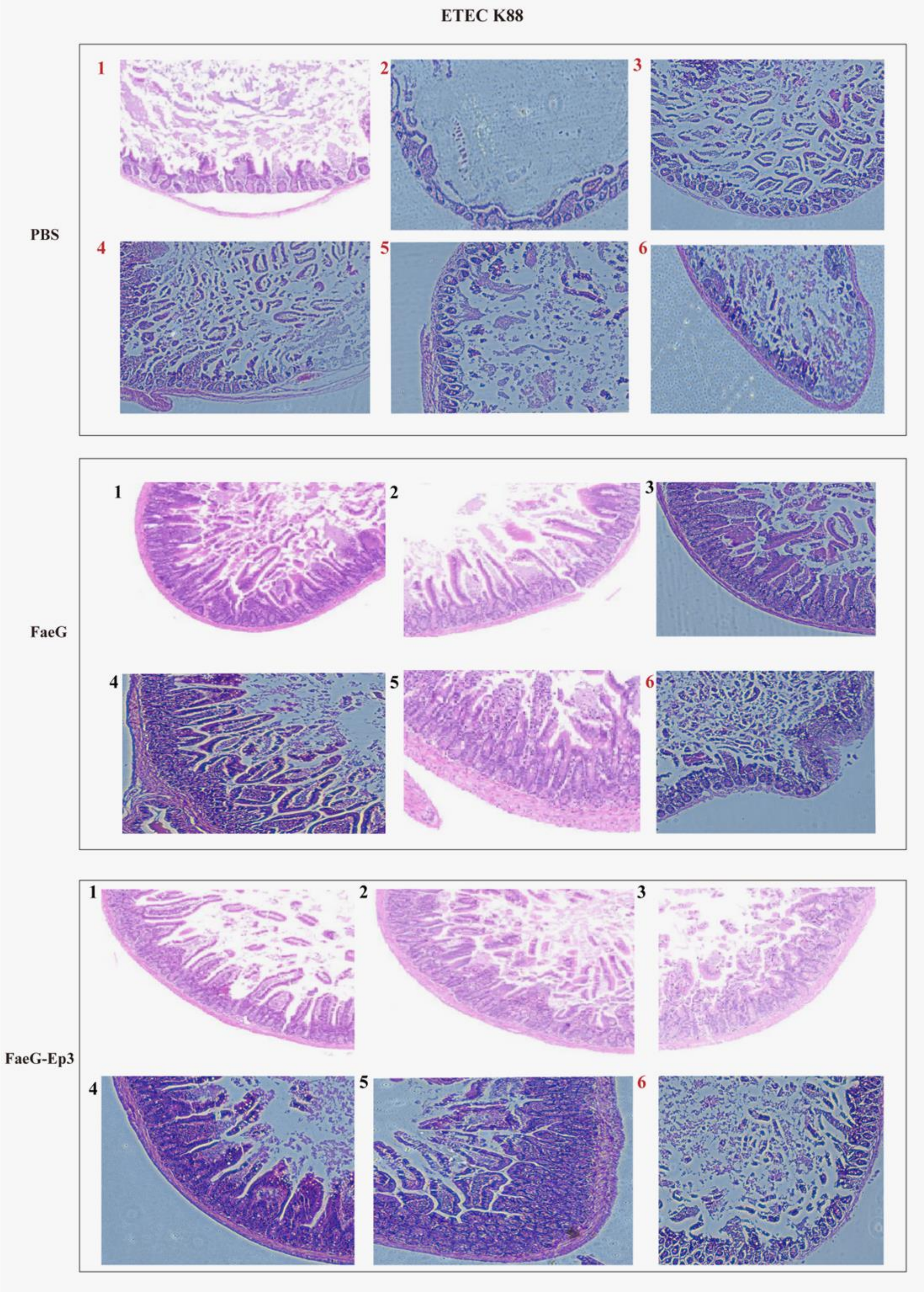

Figure S3. Histopathological analysis of the jejunum after ETEC infection.

(C) ETEC K88 infection group. bar = 100 μm

Red numbers: Villous shedding; black numbers: No villous shedding.

Supplemental Table S3. Pathological statistics after ETEC K99 infection

Group	Shedding of intestinal villi（mice）	protective rate（%）
PBS	0/6	100
ETEC K99	6/6	0
FanC	1/6	83
FaeG-Ep3	1/6	83

Supplemental Table S4. Pathological statistics after ETEC K88 infection

Group	Shedding of intestinal villi（mice）	protective rate（%）
PBS	0/6	100
ETEC K88	6/6	0
FaeG	1/6	83
FaeG-Ep3	1/6	83

.

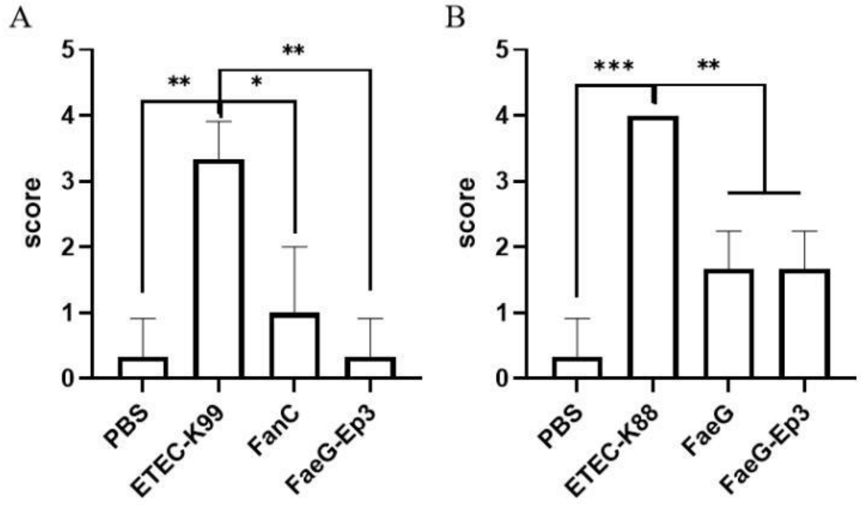

Figure S4. Double blind pathological scoring method

(A) ETEC K99 post infection score (B) ETEC K88 post infection score
