# Supplementary figures and images for: Preparation and Evaluation of Novel Epitope-Based ETEC K88-K99 Bivalent Vaccine
Source: Vet Sci. 2025 Apr 18;12(4):381. doi: 10.3390/vetsci12040381 (PMC12030781; doi:10.3390/vetsci12040381)

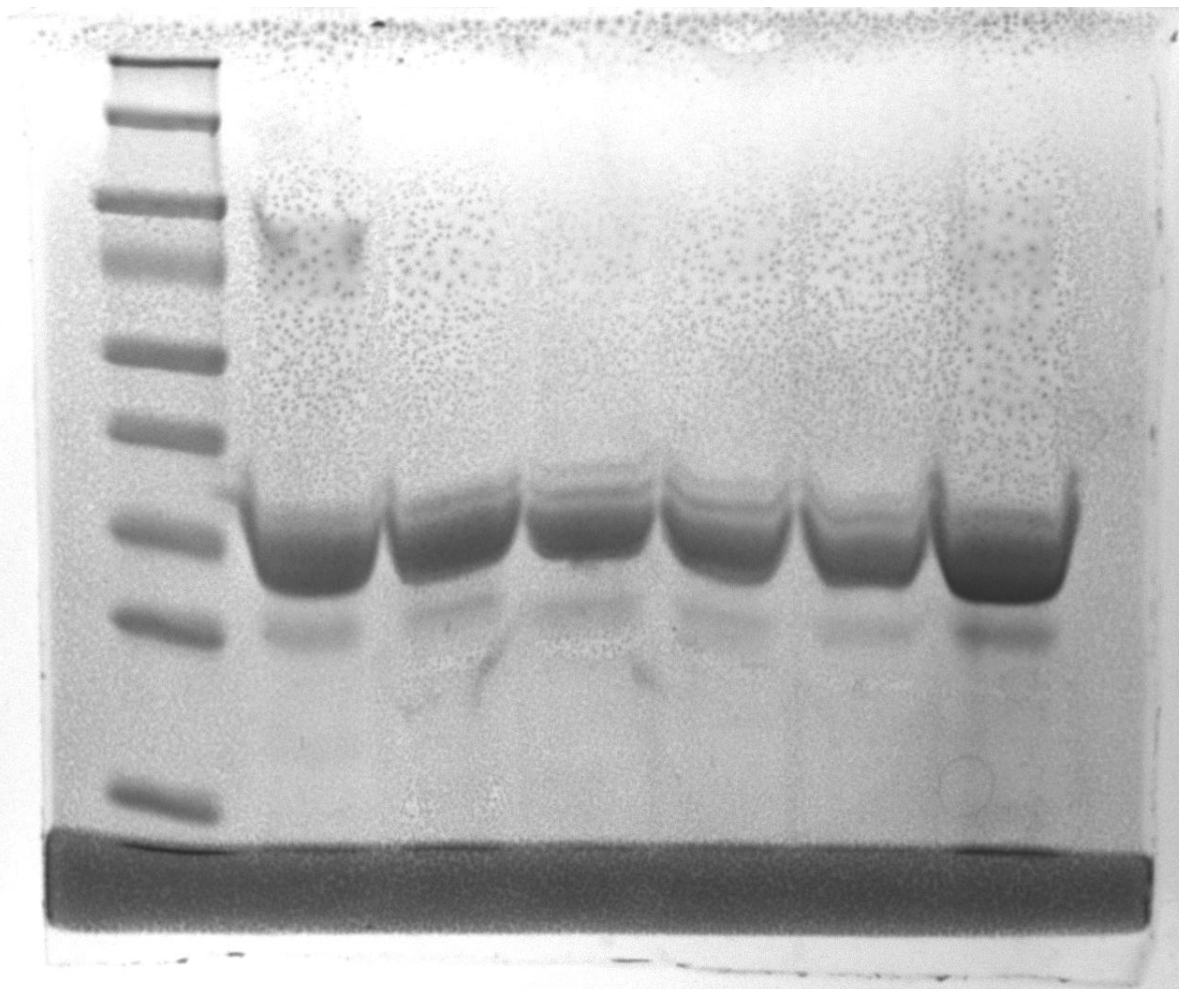

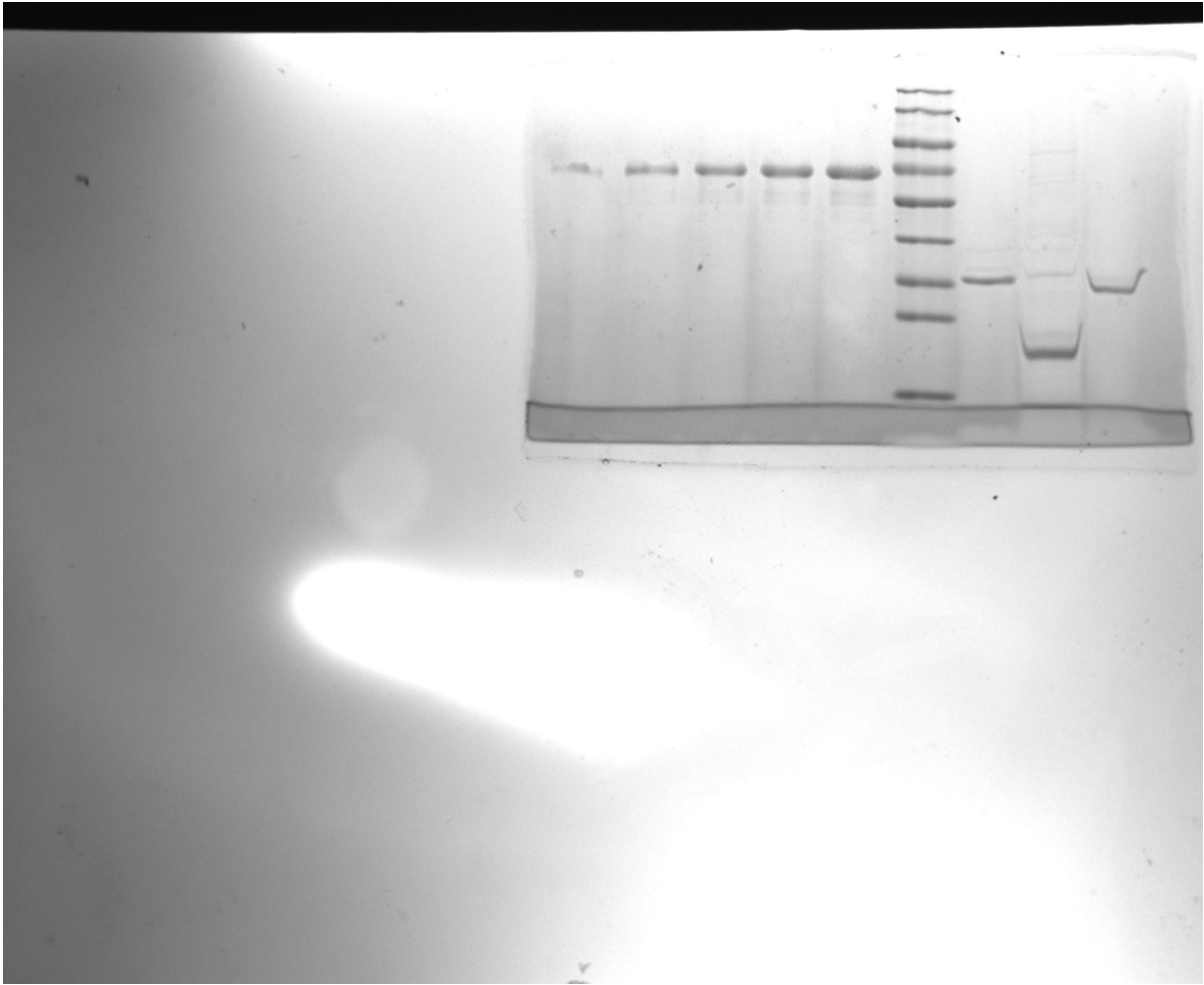

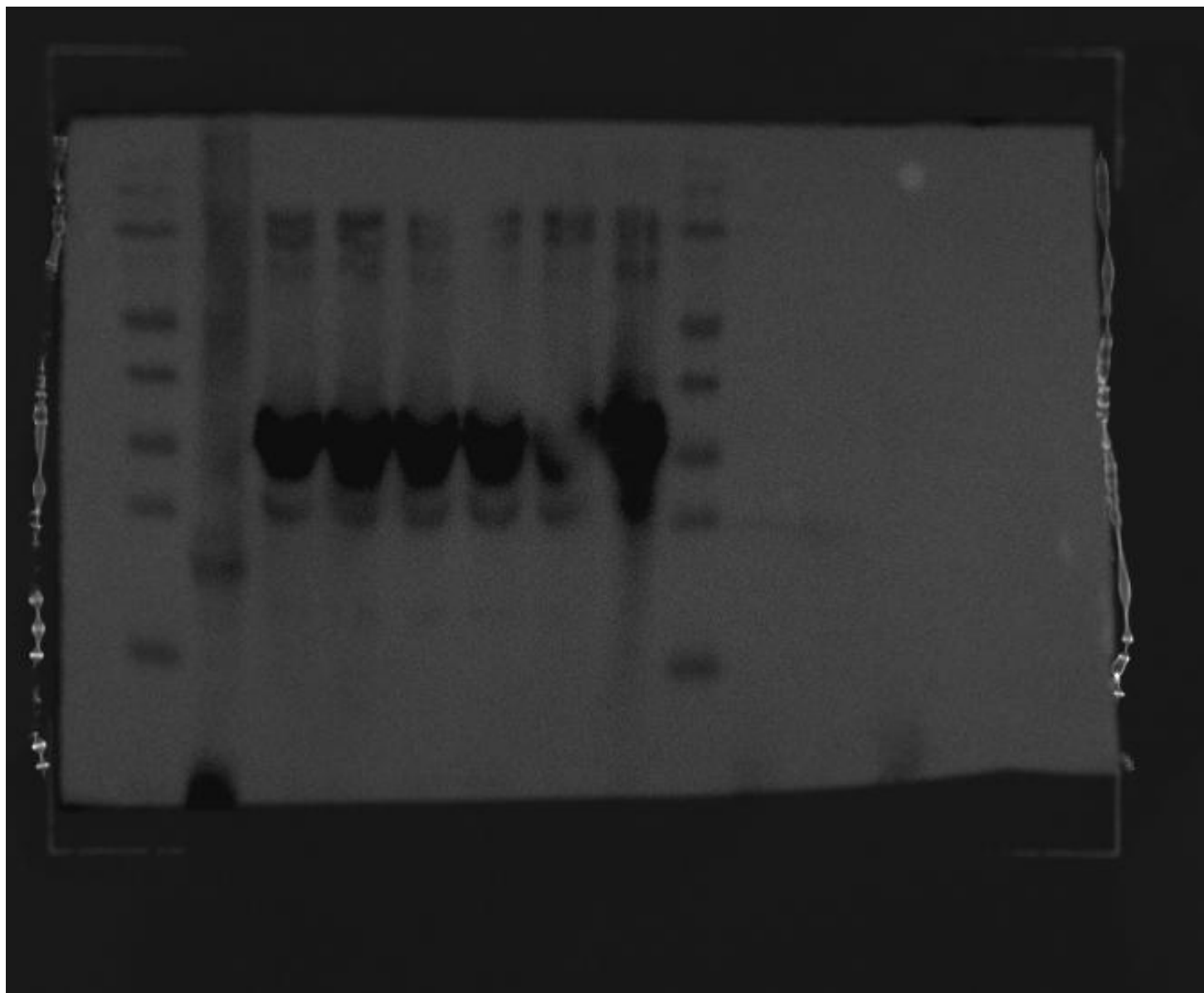

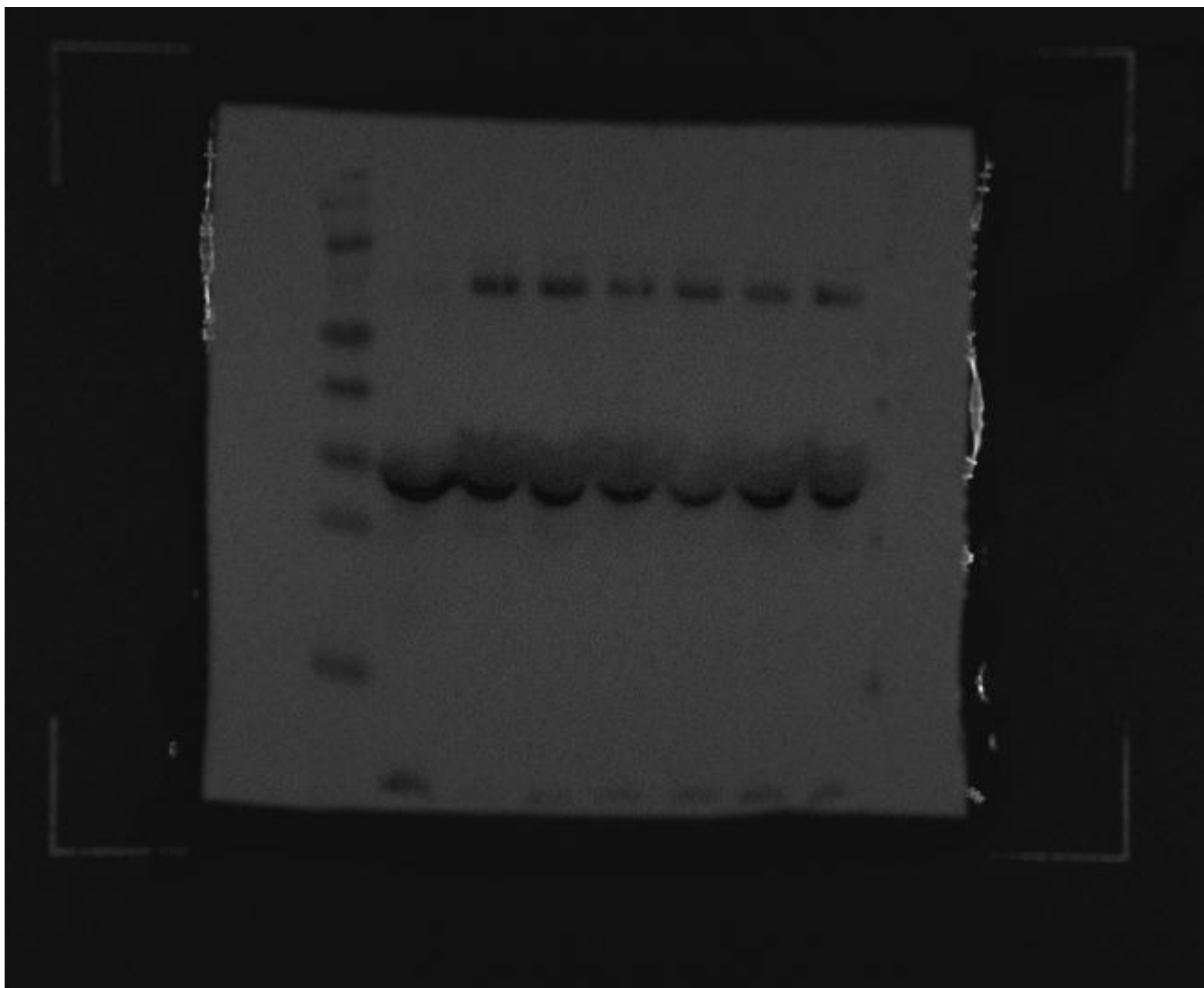

Supplement: Supplementary file 1 [file vetsci-12-00381-s001.zip › vetsci-3484386-WB.pdf]
